# Evaluation the validity and reliability of persian short form of the literacy of suicide scale (LOSS): a methodological study in 2022

**DOI:** 10.1186/s12888-023-05281-y

**Published:** 2023-10-25

**Authors:** Alireza Jafari, Mahbobeh Nejatian, Ali Mohammad Mokhtari, Fatemehzahra Naddafi, Mahdi Moshki

**Affiliations:** 1 Department of Health Education and Health Promotion, School of Health, Social Development and Health Promotion Research Center, Gonabad University of Medical Sciences, Gonabad, Iran; 2 Social Determinants of Health Research Center, Gonabad University of Medical Sciences, Gonabad, Iran; 3 Department of Epidemiology and Biostatistics, School of Health, Social Development and Health Promotion Research Center, Gonabad University of Medical Sciences, Gonabad, Iran; 4 Student Research Committee, Gonabad University of Medical Sciences, Gonabad, Iran; 5 Department of Health Education and Health Promotion, School of Health, Social Determinants of Health Research Center, Gonabad University of Medical Sciences, Gonabad, Iran

**Keywords:** Validity, Literacy of Suicide, Reliability, Psychometric, Mental, Health literacy

## Abstract

**Introduction:**

This research was conducted with the aim of evaluating the validity and reliability of Persian short version of the Literacy of Suicide Scale (LOSS) among the general population.

**Methods:**

This methodological study was conducted to evaluate the validity and reliability of Persian short form of LOSS among the general population, using a sample of 1175 participants in Iran, 2022. Participants were entered to study using method of proportional stratified sampling. The validity of Persian short form of LOSS was checked by four measures of validity: qualitative face validity, qualitative content validity, EFA (exploratory of factor analysis), and CFA (confirmatory factor analysis). The measure of reliability of Persian short form of LOSS was checked by three reliability of McDonald omega coefficient, ICC (Intraclass Correlation Coefficient), and Cronbach’s alpha coefficient.

**Results:**

Based on the findings of EFA, 4 components with eigenvalues > 1 were extracted and these 4 factors were able to explain 57.96% of variance. In EFA section, only 1 question was deleted due to entry into the non -relevant sub –scale. Based on the results of CFA, all items had the factor loading greater than 0.4 and none of the items were removed at this stage. In CFA, based the results of goodness-of-fit indexes for example: χ2/df = 2.077, NFI: 0.957, IFI = 0.977, RMSEA = 0.030, GFI: 0.988, and PGFI = 0.569, the final mode was approved with 11 items and 4 factors. In this study for all items, Cronbach’s alpha coefficient was 0.739, McDonald omega coefficient was 0.753, and ICC was 0.860. Finally, the Persian short form of LOSS was approved with 11 items and four dimensions of signs/ symptoms with 3 questions, the risk factors with 2 questions, treatment/ prevention with 2 questions, and causes/ triggers with 4 questions.

**Conclusion:**

The Persian short form of LOSS with 11 items and four subscales is a valid and reliable scale to survey the suicide literacy status in the general population.

**Supplementary Information:**

The online version contains supplementary material available at 10.1186/s12888-023-05281-y.

## Introduction

Suicide is today a serious challenge in the field of public health and is the fourth leading cause of the deaths of 15–29 years old in 2019 and one of the ten main causes of death worldwide [1]. World Health Organization reported that suicide is the cause of the death of more than 700,000 people per year; So that a suicide occurs in approximately every 40 s [1]. In Iran, the suicide death rate has increased in recent decades, with approximately 9.9 suicides per 100,000 people per year [2]. Also, the death of suicide in Iran has increased the most in recent decades among Islamic and Eastern Mediterranean countries. This reflects the inadequacy of national policies and the necessity of implementing urgent action [3].

The death of suicide will have profound effects on families and communities, and suicide will damage the community from various financial, psychological and spiritual aspects [4, 5]. If a person survives after suicide, damage to mental health, depression and suicide is not unexpected [6, 7]. On the other hand, the sadness of suicide in mourning people and families has been associated with the weakening of general health, increased physical illnesses such as cardiovascular disease, increased depression, anxiety and suicide [5, 8]. In the economic field, suicide also imposes heavy costs on the health services system of communities [9].

Suicide is a multidimensional and complex phenomenon that is affected by various cultural and social factors, genetics, childhood conditions, mental illness, a history of suicide effort, etc. [7, 10]. Although suicide is an important and preventive issue, it is usually be ignored by people due to the lack of sufficient knowledge [1]. Lack of suicide knowledge and awareness can limit the use of health care services and suicide preventive interventions at various social and individual levels [11, 12].

Suicide literacy refers to understanding the methods of prevention, symptoms and sign, causes, risk factors, and the treatment of suicide [13]. Appropriate and sufficient knowledge can facilitate the use of professional services [14]. Higher suicide literacy will lead to a decrease in suicide by encouraging people to seek out mental health services [15]. While misconceptions about the components of suicide phenomena can be directly related to the behaviors and thoughts of suicide [16, 17]. A systematic review study found that only 29.5% of those who have had suicide attempt, planning or suicide thoughts in the past year used mental health services [18]. Therefore, one of the factors that can be effective in preventing suicide is suicide literacy in the community [19, 20].

Numerous studies have shown that suicide literacy can be associated with the rates of suicide; so, higher suicide literacy will lead to a decrease in suicide by developing mental health services and reducing stigma [15, 21]. Also, the increase in suicide literacy in the community can enable them to help people who suffer from suicidal ideation and survived a suicide attempt [22]. To examine the state of suicide literacy in any population requires a valid tool in this field. According to surveys, there is no Persian version of the tool that can measure suicide literacy in the public population. One of the most comprehensive tools for examining suicide literacy is short form of literacy of suicide scale (LOSS) that designed by Calear et al. [23]. This short form of LOSS has 12 questions and four dimension of signs/ symptoms with 3 questions, the risk factors with 3 questions, treatment/ prevention with 2 questions, and causes/ triggers with 4 questions [23].

The validity and also the reliability of this scale was evaluated in different populations [15, 24–27]. Collado studied the characteristics of the Spanish version of the LOSS in the general population, and the validity and reliability of the scale was similar to other studies and was approved [28]. Han has examined the characteristics of the Chinese version of LOSS among Chinese university students [25]. The results of the study demonstrated the validity and appropriateness of the scale in assessing the consequences of suicide in the Chinese-speaking community [25]. In Iranian people, the short version of suicide literacy has not been translated and psychometric. While the short version of suicide literacy will allow researchers to measure suicidal literacy in the high samples size, and also it is easier for the participants to complete the scale. So, this research study aimed to evaluate the validity and reliability of Persian short version of LOSS among public population.

## Methods

This methodological study aimed to evaluate the validity and reliability of Persian version of short form of LOSS among 1175 public populations in Iran, in 2022.

### Sample size stage

The sources recommended that the sample size of 100, 200, 300, 500, 1000 and more are consider poor, fair, good, very well, and excellent to performing the factor analysis, respectively [29, 30]. Due to increased high danger of overfitting, it is better that the EFA (exploratory factor analysis) and CFA (confirmatory factor analysis) running on the different sample size [31]. Based on the sources, if used in the same sample for EFA and CFA, the chances of confirming the factors extracted in the EFA will increase in the CFA stage, and finally, the generalization of the CFA results will be decreased [32, 33]. In this psychometric research, EFA and CFA section were assessed by 190 participants and 1175 participants, respectively.

### Sampling method

In this research, participants were entered the study by two methods (proportional stratified sampling and simple random sampling). In first section, the number of health care centers (HC) and the population of each HC was identified and each HC was identified as a stratum. Then, in each stratum, the required samples of each stratum were selected by simple random sampling method. The inclusion criteria for selecting participants were living in the city of Gonabad, had age > 18 years old, had informed consent, and had not cognitive problem.

### Instruments for data gathering

#### 1) Demographic section

This section includes questions about demographic characteristic (such as age, marital status, sex, level of education, job status, and income status).

#### 2) Literacy of Suicide scale (LOSS)

This scale designed and evaluated by Calear et al. [23]. This scale consists of 12 questions that extracted from the long version of Literacy of Suicide Scale (LOSS-26 Items). The short form consist of four dimension of signs/ symptoms (3 questions), the risk factors (3 questions), treatment/ prevention (2 questions), and causes/ triggers (4 questions) [23]. The questions of this tool are measured by the three -choice scale of “false”, “I don’t know”, and “true”. Each item has a correct answer and the response of “I do not know” and “wrong answers” get score of zero and the correct response get score of 1. The score range of this scale is between zero and 11 and the higher score shows the proper status of suicide literacy.

### Translation process

In this section, WHO Guideline was used for translation of scale and cultural adaptation in Iran [34]. The process of cultural adaptation and translation of LOSS was performed in three phases. Before stared the process translation and back translation of the scale, the permission was obtained from main designer of LOSS. After that, in the first step, the main English version of LOSS was translated to Persian version by 2 specialists in psychology and health education and health promotion. Then two translated versions from English to Persian were compared and became one. In the second phase, the Persian version was re -translated into English and compared with the original English version. In the third phase, again the re -translated English version was translated to Persian and after the necessary modifications, the final version of the Persian scale was designed.

### Validity

In the process of examining the psychometrics of the standard questionnaire, it is not mandatory to check the quantitative face validity and quantitative content validity [35]. Suicide literacy scale is a short form standard tool, and only qualitative content and qualitative face validity were checked. To evaluate the quality face validity of Persian short form of the LOSS, use of understandable words, use of simple words, and use of a common language were checked. To evaluate the content validity of Persian short form of the LOSS, importance of each item, require time to complete tool, grammar adoption, use of appropriate words, and proper placement of each item were checked. In this study, the face validity was assessed from two points of view of the target group and the expert group. In the target group, 25 participants assessed the face validity of LOSS. In the expert group, eight specialists in psychology and health education and promotion evaluated the face validity of LOSS.

### Structure validity (EFA and CFA)

#### EFA

To performing the EFA, the SPSS V.24 software were used to check the number of basic potential component, the eigenvalues more than one, factor loading more than 0.4, scree map, and maximum 25 repetitions of rotation were used [36, 37]. To assess the appropriate sample size to performing of EFA, the Bartlett’s Test of Sphericity and KMO (Kaiser Meyer Olkin) were used [38, 39].

#### CFA

The components extracted in EFA section was surveyed in CFA using the AMOS version 24. Before evaluate the model in CFA, outlier’s data was fined by Mahalanobis statistical test and number of data were deleted. After that the data normality were assessed by two tests of kurtosis and skewness. The goodness of fit indexes of RMSEA (Root Mean Square Error of Approximation), PCFI (Parsimony Comparative Fit Index), RMR (Root Mean Square Residual), AGFI (Adjusted Goodness of Fit Index), χ2/df (Chi-Square Ratio to Degree of Freedom), GFI (Goodness of Fit Index), PNFI (Parsimonious Normed Fit Index), IFI (Incremental Fit Index), TLI (Tucker Lewis Index), CFI (Comparative Fit Index), NFI (Normed Fit Index), RFI (Relative Fit Index), and PGFI (Parsimony Goodness of Fit Index) were used for evaluating the final model [40–42]. Based on the resources, the acceptable the rate of goodness of fit indexes are RMR less than 0.08, χ2/df less than 5, AGFI more than 0.8, PGFI, PNFI, and PCFI more than 0.5, NFI, CFI, TLI, IFI, GFI, and RFI more than 0.9, and RMSEA less than 0.08 [40–43].

#### Convergent and discriminant validity

The convergent validity and discriminant validity of LOSS was evaluated using AVE (average variance extracted), MSV (maximum shared squared variance), and ASV (average shared squared variance). When AVE is greater than 0.5, convergent validity is acceptable; when both MSV and ASV are less than AVE, discriminant validity is acceptable [44].

#### Reliability stage

The scale internal consistency was evaluated by using Cronbach’s alpha coefficient and McDonald’s omega coefficient and test-retest reliability was surveyed by the ICC (Intraclass Correlation Coefficient. For the internal reliability, the ranging score between 0.70 and 0.95 is acceptable [45, 46]. In this study, 30 participants were entered in this section of study to assess the test-retest reliability (two times with a period of one month). In test-retest reliability, ICC > 0.80 is acceptable [47].

### Statistical analysis

The SPSS version 24 software were used to performed the EFA and calculated the Cronbach’s alpha coefficient and ICC. McDonald’s omega coefficient was calculated using the software JASP Version ._0.11.1_. Also, the AMOS version 24 was used for performed the CFA. In this study, the standard error of measurement (SEM = SD × √(1-ICC)) and smallest detectable change (SDC = √2 × SEM ×1.96) [48–50] were calculated for LOSS and dimensions of Causes/triggers, Risk factors, Signs, and Treatment/Prevention.

## Results

### Demographic characteristics

The mean (± standard deviation) age of people was 33.52 (± 13.04) and most of them were in age group of 18–25 years old. The majority of people were female (n = 605, 51.5%), university students (n = 453, 38.6%), and married (n = 674, 57.1%). The most people had education level of associate or bachelor’s degree (n = 586, 49.9%), and high school / diploma (n = 382, 32.5%), respectively. Other demographic characteristics were mention in Table 1.

**Table 1:** Frequency distribution of demographic characteristics (n = 1175)

Variables		N	%
**Sex**	Male	570	48.5
	Female	605	51.5
**Occupation**	Housewife	130	11.1
	University student	453	38.6
	Employed	306	26
	Retired	62	5.3
	Self-employed	174	14.8
	laborer	29	2.5
	Unemployed	21	1.8
**Marital status**	Married	674	57.1
	Single	504	42.9
**Education level**	Illiterate	2	0.2
	Elementary school	22	1.9
	Middle school	37	3.1
	High school / Diploma	382	32.5
	Associate or bachelor’s degree	586	49.9
	Master’s degree or high degree	146	12.4
**Income status**	Excellent	245	20.9
	Medium	785	66.8
	Weak	145	12.3
**Age range**	18–25	444	37.8
	26–33	237	20.2
	34–41	177	15.1
	42–49	151	12.9
	50 and more	166	14.1

### Validity assessment

Only in expert group, two items were revised and these questions were modified in terms of using simple and appropriate words.

### EFA

**Fig. 1:**
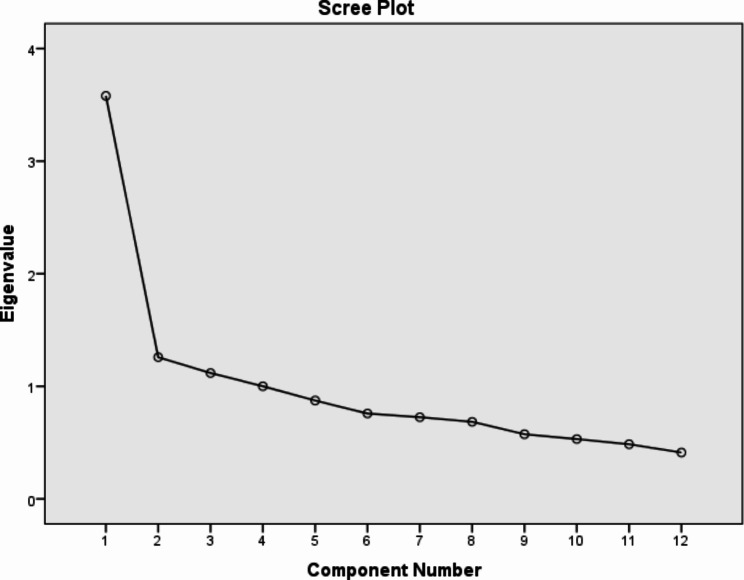
Scree plot of the factor analysis of the Persian short form of LOSS

Based on the results (KMO = 0.814, Bartlett’s test: χ2 = 422.345, df = 66, p < 0.001), sample size was good for conducting EFA. EFA showed 4 components greater one. These 4 components were able explained 57.96% of variance (Table 2; Fig. 1). In EFA section, only 1 question (“Most people who suicide are psychotic”) was deleted. In the original tool, this question is related to component of “Risk factors”, but in our study entry into component of “Treatment/Prevention” and finally deleted (Table 3).

**Table 2:** The 4 factors structure of the Persian short form of LOSS

Total Variance Explained									
Component	Initial Eigenvalues			Extraction Sums of Squared Loadings			Rotation Sums of Squared Loadings		
	Total	% of Variance	Cumulative %	Total	% of Variance	Cumulative %	Total	% of Variance	Cumulative %
1	3.580	29.830	29.830	3.580	29.830	29.830	2.180	18.163	18.163
2	1.258	10.487	40.316	1.258	10.487	40.316	1.927	16.061	34.223
3	1.118	9.313	49.630	1.118	9.313	49.630	1.436	11.967	46.191
4	1.000	8.336	57.966	1.000	8.336	57.966	1.413	11.775	57.966
5	0.874	7.280	65.245						
6	0.758	6.319	71.564						
7	0.725	6.043	77.607						
8	0.685	5.705	83.312						
9	0.574	4.784	88.097						
10	0.531	4.428	92.525						
11	0.486	4.046	96.571						
12	0.412	3.429	100.000						

Extraction Method: Principal Component Analysis.

**Table 3:** Rotated Factor Matrix of the Persian short form of LOSS

Rotated Component Matrix^a^				
Items	Component			
	Signs and symptoms	Causes/ triggers	Treatment/ Prevention	Risk factors
**S10**	**0.811**	0.127	0.115	− 0.070
**S9**	**0.738**	0.143	− 0.117	0.182
**S8**	**0.672**	0.234	0.161	0.068
**S2**	0.132	**0.761**	0.142	0.046
**S4**	0.053	**0.671**	0.079	0.056
**S1**	0.257	**0.671**	0.060	0.118
**S3**	0.382	**0.503**	0.158	0.207
**S12**	0.083	0.057	**0.829**	− 0.086
**S5**	− 0.021	0.219	**0.609**	0.259
**S11**	0.475	0.140	**0.509**	0.139
**S6**	− 0.049	0.178	0.073	**0.782**
**S7**	0.242	0.042	0.070	**0.777**

Extraction Method: Principal Component Analysis

Rotation Method: Varimax with Kaiser Normalization

a. Rotation converged in 5 iterations

### CFA

**Fig. 2:**
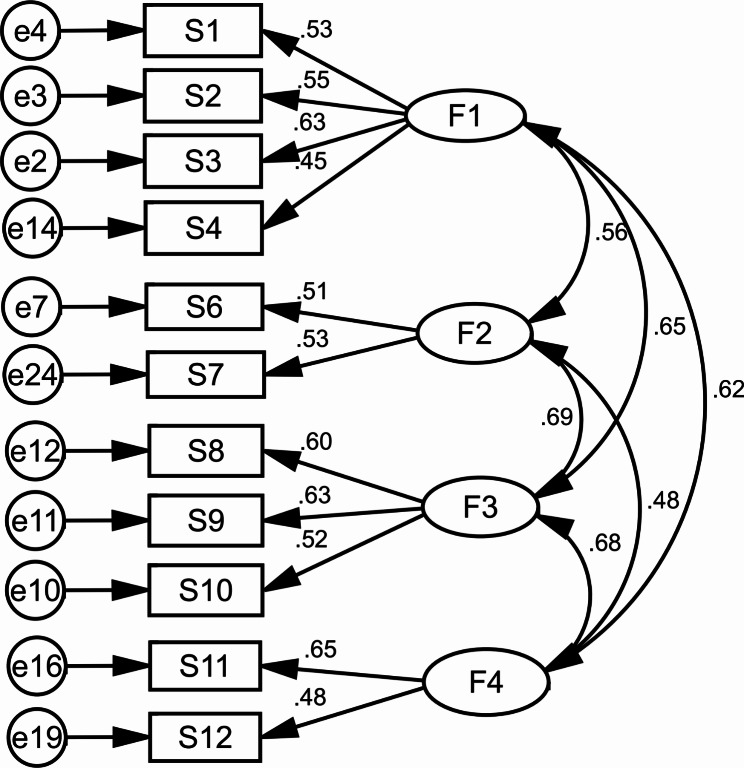
Standardized parameter estimates for the factor structure of the short form of LOSS (F1: Causes/triggers, F2: Risk factors, F3: Signs and symptoms, F4: Treatment/Prevention)

In this section all items had the factor loading greater than 0.4 and none of the items were removed at this stage (Table 4). Based the results of goodness-of-fit indexes (such as: χ2/df = 2.077, RMSEA = 0.030, GFI: 0.988, NFI: 0.957, IFI = 0.977, PGFI = 0.569, and CFI: 0.977), the final mode was approved with 11 items and 4 factors (Table 5; Fig. 2).

**Table 4:** Factor loadings of the Persian short form of LOSS

Subscales	Items	Factor loadings
**Causes/triggers**	S1. Very few people have thoughts about suicide (F)	0.533
	S2. If assessed by a psychiatrist, everyone who suicides would be diagnosed as depressed (F)	0.550
	S3. A suicidal person will always be suicidal and entertain thoughts of suicide (F)	0.630
	S4. Talking about suicide always increases the risk of suicide (F)	0.453
**Risk factors**	S5. Mos*t people who suicide are psychotic (F)*	*Deleted*
	S6. Men are more likely to suicide than women (T)	0.515
	S7. There is a strong relationship between alcoholism and suicide (T)	0.535
**Signs and symptoms**	S8. Not all people who attempt suicide plan their attempt in advance (T)	0.597
	S9. People who talk about suicide rarely kill themselves (F)	0.629
	S10. People who want to attempt suicide can change their mind quickly (T)	0.524
**Treatment/** **Prevention**	S11. People who have thoughts about suicide should not tell others about it (F)	0.651
	S12. Seeing a psychiatrist or psychologist can help prevent someone from suicide (T)	0.485

*F = False, T = True

**Table 5:** The model fit indicators of the Persian short form of LOSS

Goodness of fit indices	Confirmatory factor analysis	Standard amount of model fit indicators
**χ2**	78.934	
**df**	38	
**X** ^**2**^ **/df**	2.077	< 5
**p-value**	0.000	p > 0.05
**CFI**	0.977	> 0.9
**GFI**	0.988	> 0.9
**RMSEA**	0.030	< 0.08
**RMR**	0.016	< 0.08
**IFI**	0.977	> 0.9
**RFI**	0.937	> 0.9
**TLI**	0.966	> 0.9
**NFI**	0.957	> 0.9
**PNFI**	0.661	> 0.5
**PCFI**	0.675	> 0.5
**PGFI**	0.569	> 0.5
**AGFI**	0.979	> 0.8

### Convergent and discriminant validity

Results of Convergent and discriminant validity are visible in Table 6. In this study, Persian short form of LOSS did not have a good discriminant validity and convergent validity.

**Table 6:** Results of convergent and discriminant validity of Persian short form of LOSS

Subscales	CR	AVE	MSV	ASV
**Causes/triggers**	0.625	0.297	0.427	0.636
**Risk factors**	0.432	0.275	0.475	0.432
**Signs and symptoms**	0.608	0.342	0.475	0.613
**Treatment/Prevention**	0.490	0.330	0.457	0.511

### Reliability

For total LOSS items, the rate of Cronbach’s alpha coefficient was 0.739, McDonald omega coefficient was 0.753, and ICC was 0.860. The results of reliability of subscales of risk factors, causes/triggers, treatment/prevention, and signs and symptoms can be seen in Table 7. The standard error of measurement and smallest detectable change of LOSS were 1.367 and 3.789, respectively (Table 7). Also, the results of correlation coefficient of subscales of LOSS showed that there were a positive and significant correlation between all factors (p < 0.001). The details are visible in Table 8.

**Table 7:** Descriptive statistics of the Persian short form of LOSS

Subscales	Items	Range of score	Cronbach’s alpha coefficients	McDonald’s omega coefficients	Intraclass Correlation Coefficient (ICC)	95% Confidence Interval		P-value	SEM	SDS
						Lower Bound	Upper Bound			
**Causes/triggers**	4	0-4	0.642	0.653	0.738	0.510	0.861	< 0.001	0.967	2.680
**Risk factors**	2	0-2	0.564	0.567	0.679	0.398	0.829	< 0.001	0.696	1.929
**Signs and symptoms**	3	0-3	0.722	0.758	0.848	0.714	0.919	< 0.001	0.729	2.020
**Treatment/Prevention**	2	0-2	0.456	0.477	0.882	0.776	0.937	< 0.001	0.401	1.111
**Total of LOSS**	11	0-11	0.739	0.753	0.860	0.735	0.926	< 0.001	1.367	3.789

**Table 8:** Pearson correlation between subscales of LOSS

Subscales		Causes/ triggers	Risk factors	Signs and symptoms	Treatment/ Prevention
**Causes/triggers**	Pearson Correlation	1	0.288^**^	0.399^**^	0.352^**^
	Sig. (2-tailed)		0.000	0.000	0.000
**Risk factors**	Pearson Correlation	0.288^**^	1	0.351^**^	0.198^**^
	Sig. (2-tailed)	0.000		0.000	0.000
**Signs and symptoms**	Pearson Correlation	0.399^**^	0.351^**^	1	0.361^**^
	Sig. (2-tailed)	0.000	0.000		0.000
**Treatment/Prevention**	Pearson Correlation	0.352^**^	0.198^**^	0.361^**^	1
	Sig. (2-tailed)	0.000	0.000	0.000	

**. Correlation is significant at the 0.01 level (2-tailed).

### Ceiling and floor effect

In the present study, there was no of ceiling and floor effect, and less than 15% of respondents had the lowest score (n = 14, 1.3%) and the highest score (n = 11, 1%).

## Discussion

In this study, the 12-question of suicide literacy was evaluated in the general population of Iran. A review of available evidence shows that the validity and reliability of the short version of LOSS has not been tested in Iran. In this study, only 1 question from the Persian version of LOSS was eliminated and the modified version was confirmed with 11 items and four factors.

In our study and in the EFA stage, four components of signs/ symptoms, the risk factors, treatment/ prevention, and causes/ triggers with eigenvalue values larger than one were able to explain more than half of the variance. Also, only 1 question entitled “Most people who suicide are psychotic” was eliminated due to the entry into the unrelated component of Treatment/Prevention. Also in the CFA stage, the Factor Loading values were calculated for all questions and all of questions were larger than 0.4 and no questions were deleted at this stage. As can be seen in this study, although one question was removed from the final version, the dimensions of the main questionnaire were confirmed. The findings of this research were in line with the results of a research in China, and in the Chinese short version of LOSS one item (“Men are more likely to suicide than women”) was eliminated and the final version was confirmed with 11 questions and 4 subscales [25]. Of course, the deleted question was different from our study.

In this research, the reliability of the tool was checked using Cronbach’s alpha, Omega -McDonald’s coefficient, and ICC index, which were calculated values as 0.739, 0.753 and 0.860, respectively. The findings of this research showed that the short version of LOSS had an acceptable internal and external reliability. Based on the searches, there was no similar study on the reliability of the LOSS short version.

In another study, the psychometric characteristics of the Arabic version of the LOSS short form were examined in Jordanian students [15]. Accordingly, students had low literacy levels about suicide, and most of them had problem in questions of signs/symptoms and suicide risk factors [15]. Another study conducted in Bangladesh and among university students showed that suicide literacy in girls, medical students, people with a history of suicide in the family, and those who suffer from suicidal ideation or survived a suicide attempt was significantly higher than others [26]. As shown in the study of Calear [23], the average health literacy score in academic individuals was significantly higher than in the community. Therefore, in matters such as suicide, different groups need to be examined. Given that suicide can have different sensitivities and spreads in different groups of society (such as age and sex) in different countries, it may be better to use localized tools in each country. Therefore, based on the findings of validity and reliability of the Persian LOSS short form in this study, it seems that this questionnaire can be used to measure LOSS in the Iranian public population.

A telephone poll was conducted in Germany and the LOSS was used to measure the level of suicide literacy, which included 12 items [27]. It should be noted that in the German version, one of the questions was changed, but there was no mention of the indicators. Overall, the average suicide literacy score was 7 out of 12, indicating the average level of suicide literacy in the German population. Also, the best performance of participants related to the “treatment and prevention” area was more than 80% correct [27].

In a study conducted in Jordan’s 16-year-old Arab youth, participants were investigated using social media platforms (such as Facebook and WhatsApp) [51]. Fewer than a quarter of the participants in the study scored higher than 6 on suicide literacy, indicating low levels of suicide literacy. In general, the participants had a lot of trouble answering the questions correctly and the percentage of the correct response above 50% was not observed in any of the items [51].

The results of studies that examined the process of suicide death and death in Iran [52–54] showed an increase in suicide in the public population as well as different age groups that could confirm the need to pay attention to this issue in Iran. Therefore, given the validity and reliability of the Persian version of this tool as well as the use of this tool in other countries [15, 25–27, 51, 55], this questionnaire helped to determine the suicide literacy status of the Iranian population to take action if needed. It is suggested that future research is performed to determine the status of suicide literacy in the Iranian population with the present native questionnaire. Also, due to the necessity of evidence-based decision-making, it is recommended that the use of this native tool or the results obtained based on this tool be taken into consideration at the health management level.

### Strengths and limitation

One of limitation in this study was that the discriminant validity and convergent validity of LOSS was not good. It is recommended to test the discriminant validity and convergent validity of LOSS again in future research. In different populations and countries [52], the rate of suicide and subsequently its related factors may be different, so one of the limitations of this study is related to the generalizability of the results. In other words, considering that the present study was conducted on the general population, generalizing the validity and reliability of the questionnaire to those who are at a higher risk of suicide (such as patients with psychotic disorders) should be done with caution. On the other hand, the high sample size and the completion of the scale for different age groups and social classes is strengths of this research, while most of the previous studies were done among university students [15, 25]. Another strength of this research is that the Persian short form of LOSS takes a little time to complete the information.

## Conclusion

The Persian short form of LOSS with 11 items and four dimension of signs/ symptoms (3 questions), the risk factors (2 questions), treatment/ prevention (2 questions), and causes/ triggers (4 questions) is a good, and acceptable scale to survey the suicide literacy status in the general population. Also, given the appropriate number of questions, this questionnaire can be easily used for different people. Therefore, this questionnaire can be assisted to specify the suicide literacy status of different Iranian community and groups and provide appropriate feedback to health managers and decision makers to use appropriate interventions if necessary.

### Electronic supplementary material

Below is the link to the electronic supplementary material.

Supplementary Material 1

## Data Availability

All data generated or analysed during this study are included in this published article.

## Abbreviations

LOSS: Literacy of Suicide Scale
EFA: Exploratory factor analysis
KMO: Kaiser-Meyer-Olkin
CFA: Confirmatory factor analysis
F1: Causes/triggers
F2: Risk factors
F3: Signs and symptoms
F4: Treatment/Prevention
PCFI: Parsimony Comparative Fit Index
RFI: Relative Fit Index
GFI: Goodness of Fit Index
AGFI: Adjusted Goodness of Fit Index
IFI: Incremental Fit Index
PNFI: Parsimonious Normed Fit Index
RMSEA: Root Mean Square Error of Approximation
PGFI: Parsimony Goodness-of-Fit Index
CFI: Comparative Fit Index
x2/df: Chi-Square Ratio to Degree of Freedom
RMR: Root Mean Square Residual
TLI: Tucker Lewis Index
NFI: Normed Fit Index
SEM: Standard Error of Measurement
SDC: Smallest Detectable Change
AVE: Average Variance Extracted
MSV: Maximum Shared Squared Variance
ASV: Average Shared Squared Variance
HC: Health Care Centers
