# Supplementary material for: Evaluation the validity and reliability of persian short form of the literacy of suicide scale (LOSS): a methodological study in 2022
Source: BMC Psychiatry. 2023 Oct 25;23:783. doi: 10.1186/s12888-023-05281-y (PMC10601306; doi:10.1186/s12888-023-05281-y)
Supplement: Supplementary file 1 — Supplementary Material 1 [file 12888_2023_5281_MOESM1_ESM.docx]

**Appendix file**

**Table S1:** The final Persian short form of the Literacy of Suicide Scale (LOSS) with 11 items and four factors

| **Subscales** | **Items** |
| --- | --- |
| **F1: Causes/triggers** | 1. Very few people have thoughts about suicide (F) |
|  | 1. If assessed by a psychiatrist, everyone who suicides would be diagnosed as depressed (F) |
|  | 1. A suicidal person will always be suicidal and entertain thoughts of suicide (F) |
|  | 1. Talking about suicide always increases the risk of suicide (F) |
| **F2: Risk factors** | 1. Men are more likely to suicide than women (T) |
|  | 1. There is a strong relationship between alcoholism and suicide (T) |
| **F3: Signs and symptoms** | 1. Not all people who attempt suicide plan their attempt in advance (T) |
|  | 1. People who talk about suicide rarely kill themselves (F) |
|  | 1. People who want to attempt suicide can change their mind quickly (T) |
| **F4: Treatment/**  **Prevention** | 1. People who have thoughts about suicide should not tell others about it (F) |
|  | 1. Seeing a psychiatrist or psychologist can help prevent someone from suicide (T) |
